# The Sufferings of Christ

**Published:** 1887-08-06

**Authors:** 


### Words of Consolation.

[Communications for this column should be addressed, "Chaplain," care of the Editor of THE HOSPITAL, who will gratefully welcome any suggestions or inquiries from matrons, nurses, and the staff generally.]

### XLIV.—The Sufferings of Christ.

We find ourselves in difficulties sometimes while preparing these few "Words of Consolation," because it seems that they ought to meet the wants both of the sick and those who minister unto them. But the trials and responsibilities of these two classes in hospital, though closely allied, are very different. We have therefore often found it impossible to point our articles for both at the same time. We would gladly think, nevertheless, that nurses and attendants who find time to read to, or converse with, patients upon religious subjects may take our words as a basis for their instruction. We have already pointed out how much good may be done in this way by attendants who have within themselves sufficient spiritual life and energy to feel that their vocation in hospital is not limited simply to caring for the physical wants of the sick. To such as these we now appeal, humbly beseeching them in our Master's name to make some use, for the sake of their patients, of the articles, which will follow one another for some weeks, upon the Sufferings of Christ. We shall address them for the most part as if we were speaking to sufferers ourselves, and yet in such a manner as shall make them readable to others. We must remember (1) that after all, the very best consolation we can render to the afflicted is by endeavouring to bring them into closer touch with the Prince of Sufferers; (2) that many of our poorer brethren enter our hospitals comparative strangers to the Story of the Cross; and (3) that many more of the better instructed who have known and confessed all their lives that Jesus suffered and died to save the world, have never realised in the smallest degree with any practical faith their own personal interest in the Atonement. It is easy enough to say that Christ died for us, easy enough to believe that, as a fact in history, He really was nailed to the Cross on Calvary. But what does this amount of faith lead to in a majority of cases?—to little more than a bare, cold, intellectual assent to the truth, to no personal sympathy with the Divine Sufferer, to no distress on the part of the sinner for having caused Him so much agony, to no perception of the Cross as the measure of sin.

We appeal therefore with confidence to the spiritually minded of our readers, who have opportunities, to carry on our words, which shall be very simple, to the bedsides of patients, with a view of leading them to a better and deeper heart-knowledge of the Sufferings of Christ.

(*To be continued.*)

